# Single-Cell Pan-Cancer Atlas Reveals GPR171 as a Candidate Marker of CD8^+^ T-Cell Dysfunction

**DOI:** 10.3390/ijms27135958

**Published:** 2026-07-02

**Authors:** Xinyu Pan, Ao Zhang, Yuanyan Xiong

**Affiliations:** Key Laboratory of Gene Engineering of the Ministry of Education, School of Life Sciences, Sun Yat-sen University, Guangzhou 510275, China

**Keywords:** CD8^+^ T-cell exhaustion, tumor microenvironment, immunosuppression, immune checkpoint, *GPR171*

## Abstract

CD8^+^ T-cell exhaustion is a key mechanism of tumor immune evasion and a major limitation of current cancer immunotherapy. However, the molecular factors sustaining dysfunctional CD8^+^ T-cell states across cancers are not fully understood. Here, we identify *GPR171* as a common feature of exhausted CD8^+^ T cells across multiple solid tumors based on integrated pan-cancer single-cell transcriptomic analyses. *GPR171* is enriched in exhausted CD8^+^ T cells and is closely associated with immunosuppressive and exhaustion-related gene programs. It also shows a strong association with key immune regulatory genes such as *CTLA4* and *NR4A2*. Functional analyses suggest that reduced *GPR171* activity is associated with decreased expression of exhaustion-related genes and a shift toward cytotoxic and immune-activating programs. In parallel, a *CREM*-centered regulatory network emerges in exhausted CD8^+^ T cells and may act in concert with *GPR171*-associated programs to reinforce dysfunctional states. Overall, our results identify *GPR171* as a candidate marker of CD8^+^ T-cell dysfunction across cancers and provide a systematic pan-cancer single-cell characterization of its association with immunosuppressive T-cell states, supporting its potential as a therapeutic target for restoring antitumor immunity.

## 1. Introduction

CD8^+^ T-cell exhaustion in the tumor microenvironment (TME) is a major mechanism of tumor immune evasion. It is characterized by the progressive loss of proliferative capacity, cytotoxicity, and cytokine production under persistent antigen stimulation and immunosuppressive signaling, accompanied by sustained high expression of inhibitory receptors such as PD-1, TIM-3, LAG-3, and 2B4 [1,2]. Unlike conventional T-cell inactivation, exhaustion is a dynamic and complex differentiation process associated with extensive transcriptional, metabolic, and epigenetic remodeling [3]. Exhausted T cells are defined by sustained co-expression of multiple inhibitory receptors, including *PDCD1*, *CTLA4*, *LAG3*, *TIGIT*, and *HAVCR2* [4], together with transcriptional reprogramming mediated by *TOX* and functional remodeling characterized by expression of *ENTPD1* and *CXCL13* in tumor-infiltrating subsets [4,5]. The accumulation of exhausted CD8^+^ T cells is closely associated with poor prognosis in multiple malignancies and represents a major target of current immune checkpoint blockade (ICB) therapies [6].

G-protein-coupled receptors (GPCRs) constitute one of the largest families of cell surface receptors, and many members are critically involved in immune regulation, including chemokine receptors [7,8]. Previous studies have shown that certain GPCRs can modulate T-cell receptor (TCR) signaling and thereby influence T-cell responses [9,10]. G-protein-coupled receptor 171 (*GPR171*), a GPCR highly homologous to the P2Y receptor family [11], has been reported to participate in the regulation of feeding behavior and anxiety [12]. In addition, *GPR171* has been identified as a T-cell-associated signature gene in cancer database analyses [13,14].

Emerging evidence suggests that *GPR171* plays an important role in regulating T-cell activation and dysfunction in cancer. In melanoma, GPR171–BigLEN signaling suppresses TCR-mediated activation and limits T-cell effector function [15]. In hepatocellular carcinoma, *GPR171* has been identified as part of exhaustion-associated CD8^+^ T-cell gene signatures [16]. In recurrent malignant pleural mesothelioma, *GPR171* is enriched in exhausted CD8^+^ T cells following immune checkpoint blockade [17], suggesting a role in T-cell dysfunction and immunotherapy resistance. Although *GPR171* has been implicated in T-cell dysfunction in several cancer types, its functional role across malignancies has not been systematically characterized, leaving a critical gap in understanding its pan-cancer immunological role.

To address this knowledge gap, we investigated the role of *GPR171* in CD8^+^ T-cell dysfunction across multiple solid tumors. By integrating single-cell transcriptomic profiles from 195 samples of 96 patients spanning five cancer types, we systematically characterized the CD8^+^ T-cell compartment within the tumor microenvironment. Our analyses revealed that *GPR171* is preferentially enriched in exhausted CD8^+^ T cells and is associated with immunosuppressive transcriptional programs across cancers. Furthermore, *GPR171* is embedded within a co-expression network linked to immune checkpoint regulation and tumor-promoting pathways. Functional perturbation analysis further suggested that *GPR171* contributes to the maintenance of CD8^+^ T-cell exhaustion and immunosuppressive states. Collectively, our study identifies *GPR171* as a potential regulator and biomarker of CD8^+^ T-cell dysfunction in the tumor microenvironment.

## 2. Results

### 2.1. Pan-Cancer Single-Cell Landscape and T-Cell Distribution Across Multiple Solid Tumors

**Figure 1:**
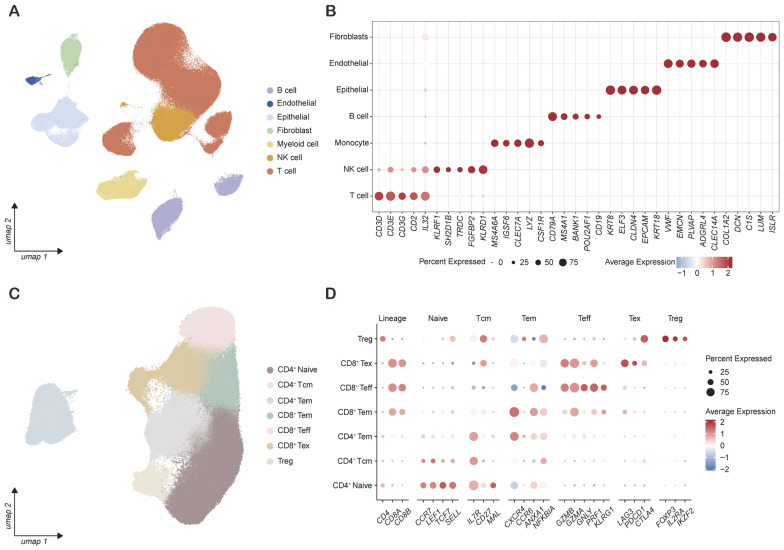
Pan-cancer single-cell transcriptomic landscape. (**A**). UMAP visualization of sc-RNA-seq data showing major cell types identified across samples, including T cells, B cells, NK cells, myeloid cells, fibroblasts, endothelial cells, and epithelial cells. (**B**). Dot plot illustrating the average expression and percent of cells expressing gene signatures across annotated cell types. (**C**). UMAP visualization of T cells showing cell types, including CD4^+^ naive T cells, CD4^+^ central memory T cells (Tcm), CD4^+^ effector memory T cells (Tem), CD8^+^ Tem, CD8^+^ effector T cells (Teff), exhausted CD8^+^ T cells (Tex), and regulatory T cells (Tregs). (**D**). Dot plot showing the average expression and percent of cells expressing gene signatures across annotated T-cell subtypes.

T cells play critical roles within the tumor microenvironment (TME), and exhaustion of CD8^+^ T cells is a hallmark of tumor immune evasion. In this study, we performed an integrative single-cell RNA sequencing (scRNA-seq) analysis on 195 samples collected from 96 patients across five solid tumor types, including colorectal cancer (CRC), esophageal carcinoma (ESCA), kidney renal clear cell carcinoma (KIRC), nasopharyngeal carcinoma (NPC), and non-small cell lung cancer (NSCLC). Samples were derived from normal tissues (N), tumor tissues (T), and peripheral blood mononuclear cells (PBMCs). Following quality control, a total of 371,007 high-quality single cells were retained for downstream clustering analysis. Initial clustering identified seven major cell populations, including T cells, B cells, NK cells, myeloid cells, fibroblasts, endothelial cells, and epithelial cells (Figure 1A,B, Supplementary Tables S1 and S2).

To further characterize the T-cell compartment, we performed refined clustering analysis, which delineated multiple T-cell subsets, including CD4^+^ naive T cells, CD4^+^ central memory T cells (Tcm), CD4^+^ effector memory T cells (Tem), CD8^+^ Tem, CD8^+^ effector T cells (Teff), exhausted CD8^+^ T cells (Tex), and regulatory T cells (Tregs) (Figure 1C,D, Supplementary Table S3). Among the five cancer types analyzed in this study, CRC exhibited marked heterogeneity in T-cell distribution, whereas the other four cancer types showed relatively uniform distribution patterns (Figure S1A). At the subtype level, T cells in CRC were predominantly composed of CD4^+^ Tem cells, while CD4^+^ Tcm cells were mainly enriched in NSCLC tissues. In contrast, CD8^+^ T-cell subsets displayed a relatively even distribution across all cancer types (Figure S1B).

### 2.2. GPR171 Represents a Potential Marker of CD8^+^ T-Cell Exhaustion

**Figure 2:**
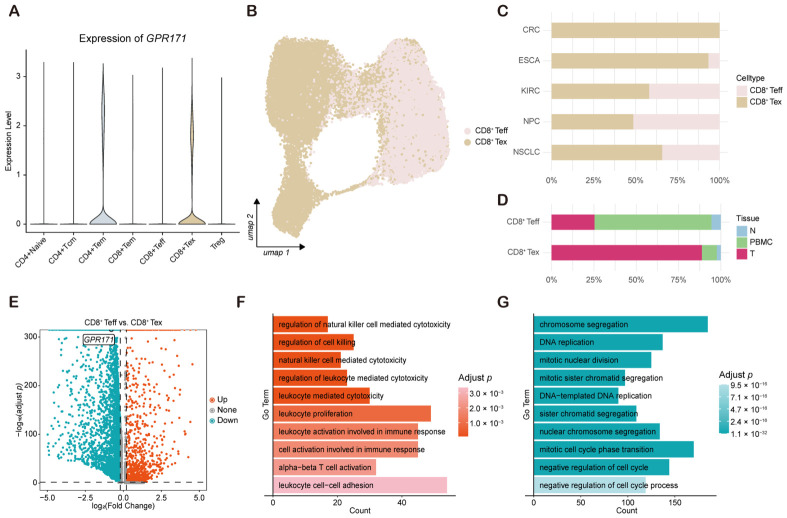
Expression and functional analysis of GPR171 in CD8^+^ T-cell subsets. (**A**). Vlnplot showing the expression of *GPR171* across T-cell subsets. (**B**). UMAP projection showing the distribution of CD8^+^ Tex and CD8^+^ Teff cells. (**C**). Bar plot showing the proportions of CD8^+^ Tex and CD8^+^ Teff cells across different tumor types. (**D**). Bar plot illustrating the tissue distribution of CD8^+^ Tex and CD8^+^ Teff cells. (**E**). Volcano plot showing differentially expressed genes between CD8^+^ Tex and CD8^+^ Teff cells, with *GPR171* highlighted. (**F**). GO enrichment analysis of upregulated genes in CD8^+^ Teff cells. (**G**). GO enrichment analysis of upregulated genes in CD8^+^ Tex cells.

To investigate the potential involvement of *GPR171* in T-cell functional regulation, we systematically evaluated its expression across distinct T-cell subsets. *GPR171* expression was significantly elevated in CD4^+^ Tem and CD8^+^ Tex cells relative to other T-cell populations (Figure 2A). However, given that the CD4^+^ Tem population was predominantly derived from CRC samples, its expression pattern may not accurately reflect a pan-cancer trend. By contrast, the marked differential expression of *GPR171* between CD8^+^ Teff and CD8^+^ Tex cells suggested a potential association between *GPR171* and CD8^+^ T-cell exhaustion across multiple cancer types. Accordingly, subsequent analyses were focused on the CD8^+^ Teff and CD8^+^ Tex subpopulations (Figure 2B).

When stratified by cancer type, both CD8^+^ Teff and CD8^+^ Tex cells were identified across all tumor types except CRC, with relatively homogeneous distributions observed in KIRC, NPC, and NSCLC (Figure 2C, Supplementary Table S1). Due to the insufficient numbers of CD8^+^ Teff and CD8^+^ Tex cells in CRC samples, all subsequent analyses were performed using the other four cancer types. Stratification according to tissue source demonstrated that CD8^+^ Teff cells were predominantly enriched in peripheral blood and adjacent normal tissues (74.4%), whereas only a minor fraction was detected within tumor tissues. In contrast, CD8^+^ Tex cells were primarily localized within tumor tissues, accounting for 89.4% of the total CD8^+^ Tex population (Figure 2D). Differential expression analysis further confirmed that *GPR171* expression was significantly upregulated in CD8^+^ Tex cells compared with CD8^+^ Teff cells (log2FC = −0.790, adjusted *p*-value = 0) (Figure 2E).

Gene Ontology (GO) enrichment analysis revealed that genes upregulated in CD8^+^ Teff cells were primarily associated with immune effector functions, including regulation of cell killing (adjust *p*-value = 1.0 × 10^−4^), natural killer cell mediated cytotoxicity (adjust *p*-value = 1.1 × 10^−4^), leukocyte proliferation (adjust *p*-value = 7.4 × 10^−4^), and leukocyte activation involved in immune response (adjust *p*-value = 1.4 × 10^−3^) (Figure 2F). In contrast, genes preferentially expressed in CD8^+^ Tex cells exhibited enrichment in pathways associated with cell cycle inhibition (negative regulation of cell cycle, adjusted *p*-value = 1.7 × 10^−17^; negative regulation of cell cycle process, adjusted *p*-value = 9.5 × 10^−16^), while lacking enrichment for canonical immune activation programs (Figure 2G). These transcriptional characteristics are consistent with the established molecular features of exhausted CD8^+^ T cells [3,4,5], thereby further supporting the potential utility of *GPR171* as a candidate pan-cancer biomarker of T-cell exhaustion.

### 2.3. GPR171 in a CD8^+^ T-Cell Immunosuppressive Co-Expression Network

**Figure 3:**
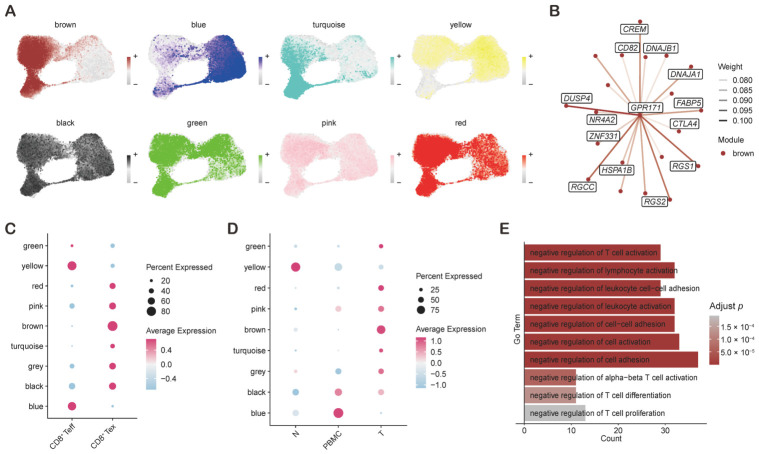
hdWGCNA analysis reveals an immunosuppressive transcriptional program. (**A**). Feature plots showing the expression patterns of each module in CD8^+^ Tex and CD8^+^ Teff cells, excluding the gray (noise) module. (**B**). Weighted gene co-expression network illustrating genes strongly co-expressed with *GPR171*. (**C**). Dot plot showing the average expression and percentage of cells expressing each module in CD8^+^ Tex and CD8^+^ Teff cells. (**D**). Dot plot showing the average expression and percentage of cells expressing each module across T cells from different tissue origins. (**E**). GO-BP enrichment analysis of the brown module.

To elucidate co-expression patterns associated with *GPR171*, we performed high-dimensional weighted gene co-expression network analysis (hdWGCNA) in CD8^+^ Teff and CD8^+^ Tex cells. The soft-thresholding power was selected according to the default criteria recommended by the hdWGCNA framework (Figure S2A), resulting in the identification of eight distinct co-expression modules (Figure 3A and Figure S2B), each assigned a unique color for visualization purposes. *GPR171* was found to be clustered within the brown module.

Network analysis of the brown module revealed strong co-expression relationships among *GPR171*, *CTLA4*, *FABP5*, *DUSP4*, and *NR4A2* (Figure 3B). Notably, *CTLA4* is a well-established immune checkpoint molecule that plays a central role in negative regulation of T-cell activation [18]. *FABP5* has been implicated in tumor progression through modulation of lipid metabolism [19], whereas *DUSP4* is associated with MAPK signaling and p53 pathways, contributing to tumor cell proliferation and survival [20]. *NR4A2* has also been reported to be closely linked to tumor progression and malignancy [21]. Importantly, these genes were identified as hub genes within the brown module, suggesting that this module is functionally associated with pro-tumorigenic activity. These findings suggest that the brown module represents an immunosuppressive and pro-tumorigenic network integrating immune checkpoint regulation, metabolic reprogramming, and pro-survival signaling pathways that may contribute to tumor progression.

At the expression level, the brown module exhibited the strongest association with CD8^+^ Tex cells, while showing the lowest expression in CD8^+^ Teff cells (Figure 3C). In addition, this module was predominantly enriched in tumor tissues, with minimal expression observed in normal tissues and PBMC (Figure 3D). Functionally, the brown module was significantly enriched in immunosuppressive biological processes, including inhibition of T-cell activation, lymphocyte activation, and leukocyte activation, as well as suppression of T-cell differentiation and proliferation (Figure 3E). Collectively, these findings indicate that the brown module represents a tumor-promoting, immunosuppressive transcriptional program, within which *GPR171* is embedded as a key component.

### 2.4. GPR171 Is Associated with CD8^+^ T-Cell Dysfunction

**Figure 4:**
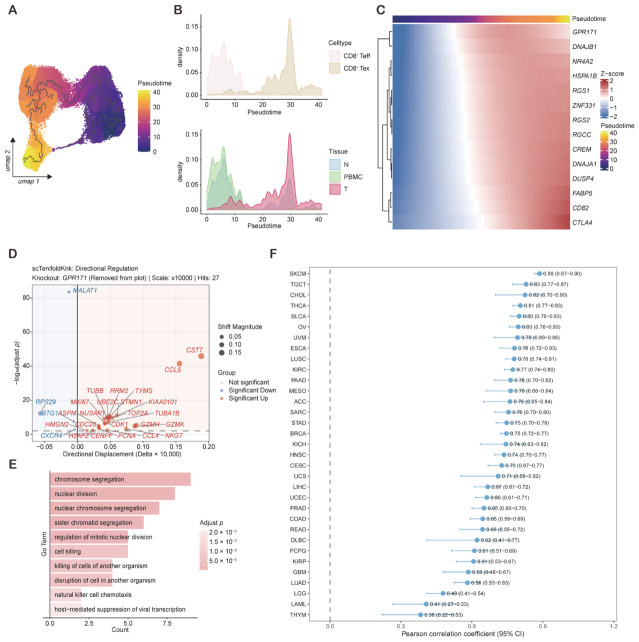
Trajectory analysis and virtual gene knockout reveal that *GPR171* suppresses T cell immune function. (**A**). UMAP pseudotime plot showing inferred developmental trajectories with pseudotime progression indicated by a color gradient. (**B**). (**Top**) Density plots comparing the distribution of CD8^+^ Tex and CD8^+^ Teff cells along pseudotime. (**Bottom**) Density plots comparing the distribution of T cells from different tissue origins along pseudotime. (**C**). Heatmap depicting the expression dynamics of genes from the immunosuppressive transcriptional program along the pseudotime trajectory. (**D**). Volcano plot showing differential gene expression changes induced by virtual knockout of *GPR171* using scTenifoldKnk, with genes shifted upon perturbation highlighted. (**E**). GO-BP enrichment analysis of genes upregulated after *GPR171* virtual knockout. (**F**). Correlation between *GPR171* expression and T-cell exhaustion signature scores across multiple cancer types. Pearson correlation coefficients are shown on the x-axis. Dots represent correlation estimates and horizontal bars indicate 95% confidence intervals. Cancer types from TCGA are ordered by correlation strength.

To reveal the gene expression dynamics underlying T-cell exhaustion, we performed pseudotime trajectory analysis of CD8^+^ Teff and CD8^+^ Tex cells (Figure 4A). The results demonstrated that CD8^+^ Teff cells were predominantly distributed at the early stages of the trajectory, whereas CD8^+^ Tex cells were mainly enriched in the intermediate and late stages. Consistently, cells derived from normal tissues and PBMC were largely mapped to the early trajectory state in a manner similar to CD8^+^ Teff cells, whereas cells from tumor tissues aligned predominantly with the CD8^+^ Tex-associated late trajectory state (Figure 4B), suggesting that progression along the trajectory is closely associated with CD8^+^ T-cell exhaustion. In addition, genes constituting the previously identified tumor-promoting and immunosuppressive transcriptional program exhibited low expression in early trajectory states and progressively increased expression toward late stages (Figure 4C), indicating a strong association between this gene program and the dynamic progression of CD8^+^ T-cell exhaustion.

To further investigate the functional role of *GPR171* in CD8^+^ T cells, we performed a scTenifoldKnk-based virtual gene knockout analysis of *GPR171* in CD8^+^ Tex cells. The results showed that depletion of *GPR171* led to decreased expression of *CXCR4*, a gene previously reported to promote T-cell exhaustion [22] (Figure 4D). Conversely, genes upregulated following *GPR171* knockout were functionally enriched in pathways related to cell proliferation, cell killing, and NK cell chemotaxis (Figure 4D). In additional sub-sample validation analyses, consistent patterns were observed across cohorts. In the ESCA cohort, genes upregulated following *GPR171* knockout were predominantly enriched in pathways associated with cell proliferation (Figure S3A,B). Similarly, in the NPC cohort, *GPR171* depletion also resulted in upregulation of genes mainly involved in cell proliferation, as well as pathways related to cell killing and NK cell-mediated immune functions (Figure S3C,D). Collectively, these computational findings suggest that *GPR171* may act as a negative regulator of CD8^+^ T-cell function, potentially suppressing T-cell proliferation, and highlight its value as a putative therapeutic target in tumor immunity.

In pan-cancer analysis across 33 TCGA cancer types, *GPR171* expression showed a broadly positive correlation with the T-cell exhaustion GSVA score, with varying effect sizes across tumor types. The strongest associations were observed in SKCM (r = 0.89, 95% CI: 0.87–0.90), TGCT (r = 0.83, 95% CI: 0.77–0.87), and CHOL (r = 0.82, 95% CI: 0.70–0.90), as well as multiple other solid tumors including THCA, BLCA, and OV (r ≈ 0.80). Moderate positive correlations were also detected in ESCA (r = 0.78, 95% CI: 0.72–0.83) and most remaining cancer types. In contrast, relatively weaker associations were observed in hematological and select solid tumors, such as LAML (r = 0.41, 95% CI: 0.27–0.53) and THYM (r = 0.36, 95% CI: 0.22–0.53) (Figure 4F, Supplementary Table S4). Overall, these results indicate a consistent pan-cancer trend in which higher *GPR171* expression is associated with elevated T-cell exhaustion signatures, suggesting a consistent computational association between *GPR171* expression and T-cell exhaustion signatures across diverse tumor contexts.

### 2.5. CREM-Associated Regulatory Network in CD8^+^ T-Cell Exhaustion

**Figure 5:**
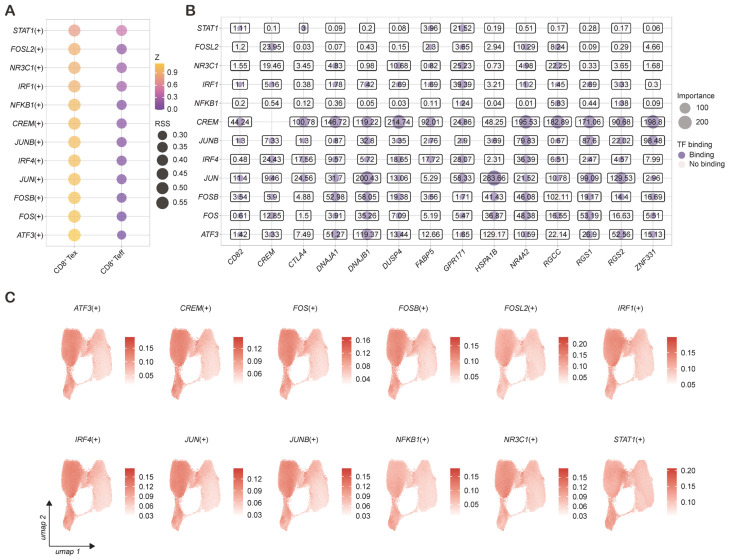
pySCENIC analysis identifies transcription factor networks regulating *GPR171* expression. (**A**). Comparison of transcription factor activity scores between the CD8^+^ Tex and CD8^+^ Teff groups using pySCENIC, with dot size representing the regulon specificity score (RSS) and color indicating the Z score. (**B**). Regulatory association analysis between transcription factor expression and expression levels of genes in the immunosuppressive transcriptional program. Dot size represents the inferred regulatory importance score from pySCENIC GRNBoost2 network inference. (**C**). Distribution of regulon AUC scores for 12 candidate transcription factors associated with the immunosuppressive transcriptional program in CD8^+^ Tex and CD8^+^ Teff cells.

To identify the transcription factor network regulating CD8^+^ T-cell exhaustion, we used pySCENIC to predict transcription factor activity from scRNA-seq data of CD8^+^ Teff and CD8^+^ Tex cells. This analysis identified 12 potential transcription factors associated with *GPR171*, most of which exhibited stronger activity in CD8^+^ Tex cells (Figure 5A), consistent with the aforementioned results. We found that *JUN* showed the strongest association with *GPR171* expression. However, despite this strong association, *JUN* showed limited connections with the broader immunosuppressive transcriptional program. In contrast, *CREM* showed a strong regulatory association with *GPR171* and even stronger regulatory relationships with *CTLA4*, *FABP5*, *DUSP4*, and *NR4A2* (Figure 5B). We further examined the regulon activity (AUCell) of these transcription factors and found that most were enriched in CD8^+^ Tex cells, showing higher regulon activity and increased cellular abundance within this subset (Figure 5C). Among them, *CREM* exhibited a highly consistent expression pattern, with marked overlap with CD8^+^ Tex cells. Previous studies have reported that *CREM* mediates suppression of NK cell antitumor activity via the PKA–CREB signaling pathway, and that *CREM* knockout significantly enhances the cytotoxicity and antitumor efficacy of CAR-NK cells [23]. Collectively, our findings suggest that the *CREM*-associated regulatory network is closely associated with the CD8^+^ T-cell exhaustion program and provides a computationally inferred regulatory landscape underlying this cellular state.

## 3. Discussion

CD8^+^ T-cell exhaustion is a major mechanism of tumor immune evasion and is characterized by progressive loss of effector function accompanied by extensive transcriptional reprogramming [24,25]. Although immune checkpoint blockade has significantly improved cancer treatment outcomes, many patients still fail to achieve durable responses [26,27], highlighting the need to identify additional regulators of T-cell dysfunction. In this study, we identified *GPR171* as a potential pan-cancer marker associated with exhausted CD8^+^ T cells across multiple solid tumors.

Our results demonstrated that *GPR171* was preferentially enriched in CD8^+^ Tex cells and predominantly localized within tumor tissues, suggesting a close association with the immunosuppressive tumor microenvironment. In contrast, CD8^+^ Teff cells were mainly enriched in peripheral blood and normal tissues and retained immune activation signatures. Co-expression analysis further revealed that *GPR171* was embedded within an immunosuppressive transcriptional program containing multiple tumor-promoting and exhaustion-associated genes, including *CTLA4*, *FABP5*, *DUSP4*, and *NR4A2* [18,19,20,21]. These findings suggest that *GPR171* may participate in coordinated regulatory networks associated with T-cell dysfunction within the tumor microenvironment.

Importantly, trajectory analysis indicated that *GPR171*-associated transcriptional programs progressively increased during the transition from effector to exhausted CD8^+^ T-cell states. In addition, virtual knockout of *GPR171* reduced *CXCR4* expression while restoring transcriptional programs associated with cytotoxicity and immune activation, supporting a potential association between *GPR171* expression and exhausted T-cell states. Consistent with our findings, previous studies have demonstrated that interaction between *GPR171* and its neuropeptide ligand BigLEN suppresses T-cell receptor-mediated signaling and inhibits T-cell activation and proliferation. In melanoma models, *GPR171* deficiency enhanced antitumor immunity, while pharmacological inhibition of *GPR171* significantly promoted antitumor T-cell responses [15]. Moreover, combined treatment with a *GPR171* antagonist and anti-PD-1 monoclonal antibody markedly suppressed tumor growth and improved survival in the MC38 tumor model [15]. In hepatocellular carcinoma, *GPR171* has also been identified as a component of exhaustion-associated CD8^+^ T-cell gene signatures [16]. Furthermore, exhausted CD8^+^ T cells expressing *GPR171* were observed in recurrent malignant pleural mesothelioma following immune checkpoint blockade, suggesting a potential association between *GPR171* and acquired immunotherapy resistance [17].

Furthermore, we identified a *CREM*-associated transcriptional regulatory network enriched in CD8^+^ Tex cells. Notably, *CREM* exhibited strong regulatory connectivity with *GPR171* and multiple immunosuppressive hub genes, suggesting that *CREM*- and *GPR171*-associated regulatory programs may be linked to immunosuppressive T-cell states in tumors.

Overall, our findings identify *GPR171* as a potential biomarker and regulatory component of CD8^+^ T-cell dysfunction across multiple cancers. These results provide new insights into the molecular features associated with T-cell exhaustion and suggest that targeting *GPR171*-associated immunosuppressive programs may represent a promising strategy for cancer immunotherapy.

## 4. Materials and Methods

### 4.1. Data Collection

Single-cell transcriptomic data were retrieved from the National Center for Biotechnology Information (NCBI) Gene Expression Omnibus (GEO; accession number: GSE160269, GSE121636, GSE166555, GSE162025 and GSE162498) [28,29,30,31,32]. To control for the imbalance in cell numbers between tumor and normal tissues and its potential impact on downstream analyses, random downsampling was performed on the group with a larger number of cells to match the cell count of the other group. The random sampling was conducted in the R environment, and reproducibility was ensured by setting the random seed to set.seed(123). Metadata fields were harmonized to ensure consistent annotation across all datasets. We obtained RNA-seq count data and clinical information for 11,236 samples across 33 cancer types from the GDC Hub of UCSC Xena https://xenabrowser.net/datapages/ (accessed on 1 December 2024).

### 4.2. Normalization, Feature Selection, Scaling, Dataset Integration, and Batch Effect Correction

All datasets were integrated and processed using Seurat (v5.0.3) [33]. First, datasets were normalized using the NormalizeData function, and the top 3000 highly variable genes (HVG) were identified using the FindVariableFeatures function. Data were scaled with the ScaleData function, followed by dimensionality reduction performed using the RunPCA function. Subsequently, all datasets were merged into a single Seurat object, with redundant assays and metadata discarded. The integrated dataset was subjected to reprocessing, including normalization, variable gene selection, scaling, and PCA. Batch effects were corrected using Harmony (v1.2.0) [34], with sample ID designated as the batch variable.

### 4.3. Cell Type Annotation and Marker Gene Identification

Cell type annotation was performed using SingleR (v2.2.0) [35], with clusters annotated to major cell types based on the tool’s output, using the Human Primary Cell Atlas reference dataset (HumanPrimaryCellAtlasData). Cell type-specific marker genes were identified using COSG (v0.9.0) [36], and the top 50 marker genes per cell type were selected based on the RNA assay and log-normalized expression values. In addition, canonical marker genes for each lineage were curated based on prior biological knowledge. Both the automated annotations and identified marker gene were further refined by manual inspection. Cell type assignments were corrected as necessary according to marker gene expression profiles, ensuring maximal annotation accuracy.

### 4.4. Differential Gene Expression (DEGs) and GO Enrichment

DEGs between groups (e.g., treatment efficacy, cell type, or state) were identified using Seurat’s FindMarkers function, with the Wilcoxon rank-sum test and false discovery rate (FDR) correction applied. Genes with adjusted *p*-value < 0.01 and |avg_log2FC| > 0.2 were considered significant. GO Biological Process (BP) functional enrichment analysis was conducted using clusterProfiler (v4.2.2) [37].

### 4.5. Weighted Gene Co-Expression Network Analysis (hdWGCNA)

To explore gene co-expression modules, hdWGCNA (v0.3.03) [38] was applied to selected cell subsets (e.g., CD8^+^ T-cell subclusters). Genes expressed in >1% of cells were used to construct metacells, followed by data normalization and construction of signed co-expression networks with a soft-thresholding power of 7 and subsequent Topological Overlap Matrix (TOM) calculation.

### 4.6. Trajectory and Pseudotime Analysis

Pseudotime trajectories were constructed using Monocle3 (v1.4.26) [39,40,41]. Dimensionality reduction was first performed, followed by batch effect correction using a mutual nearest neighbor (MNN)-based approach [42]. Cell trajectory structures were then reconstructed in the low-dimensional space, and cells were ordered along pseudotime. By integrating the expression profiles of key genes, their dynamic changes during pseudotime progression were analyzed to elucidate disease-associated transcriptional regulatory processes.

### 4.7. Gene Regulatory Network Inference (pySCENIC)

Gene regulatory networks (GRNs) were reconstructed using pySCENIC (v0.12.1) [43]. Cell-level expression matrices were exported, and GRNs were inferred using the GRNBoost2 algorithm. This was followed by motif enrichment analysis and regulon activity (AUCell) score.

### 4.8. Single-Cell Virtual Gene Knockout (scTenifoldKnk)

Single-cell virtual perturbation analysis was performed using scTenifoldKnk (v 1.0.3) [44]. Cells with non-zero expression of the target gene were selected for downstream analysis. Gene expression matrices were normalized by library size scaling followed by log-transformation prior to analysis. Directionality was defined based on the displacement of the target gene in the manifold alignment space, which was used to set a reference direction for subsequent interpretation of regulatory effects.

### 4.9. GPR171 Expression Correlation with T Cell Exhaustion Scores Using ssGSEA in TCGA Cohorts

T-cell exhaustion signature scores were computed using a curated exhaustion gene set (*PDCD1*, *CTLA4*, *HAVCR2*, *TIGIT*, *LAG3*, *TOX*, *ENTPD1*, and *CXCL13*) [4,5]. For each TCGA cohort, gene expression matrices were first log2-transformed (log2 [count + 1]) and gene symbols were mapped from Ensembl IDs using org.Hs.eg.db (v3.22.0). Single-sample gene set enrichment analysis (ssGSEA) was then performed using the GSVA (v2.4.0) [45] to quantify exhaustion-related pathway activity for each sample. Pearson correlation analysis was applied to evaluate the association between GPR171 expression and exhaustion scores across samples within each cancer type, and 95% confidence intervals were estimated using Fisher’s z-transformation. Statistical significance was assessed using two-sided tests.

### 4.10. Visualization

Visualization was performed using the following software packages: Seurat (v5.0.3), Monocle3 (v1.4.26), ggplot2 (v3.4.4), cowplot (v1.1.1), ggpubr (v0.6.0), ggrepel (v0.9.4), pheatmap (v1.0.12) and GOplot (v1.0.2) in R.

### 4.11. Statistics and Reproducibility

Statistical significance was defined as *p*-value < 0.05. All statistical analyses were performed in R (v4.5) environment with RStudio (v 2022.12.0+353, “Elsbeth Geranium” Release). For single-cell data, DEG analyses were performed using the Wilcoxon rank-sum test.

## 5. Conclusions

This study identifies *GPR171* as a candidate marker of exhausted CD8^+^ T cells across multiple solid tumors and demonstrates its preferential enrichment in tumor-infiltrating CD8^+^ Tex cells. *GPR171* is embedded within immunosuppressive transcriptional programs and is associated with progressive T-cell dysfunction during the transition from effector to exhausted states. Network analysis further reveals a *CREM*-associated regulatory axis linked to *GPR171* and key immunosuppressive genes, suggesting coordinated transcriptional control of T-cell exhaustion. Functional inference indicates that *GPR171*-associated programs are linked to a broad suppression of cytotoxic and immune activation signatures, consistent with a role in sustaining dysfunctional T-cell states. Collectively, our findings implicate *GPR171* as a key component of exhaustion-associated regulatory networks and highlight its potential as a biomarker and therapeutic target for restoring anti-tumor immunity in cancer immunotherapy.

## Data Availability

Data included in this paper were acquired from the National Center for Biotechnology Information (NCBI) Gene Expression Omnibus (GEO; accession number: GSE160269, GSE121636, GSE166555, GSE162025 and GSE162498). No algorithm or software was generated for this study. The workflow code employed in this study is available in the Zenodo repository at the following DOI: 10.5281/zenodo.20368371. The repository contains the scripts necessary to reproduce the analyses conducted in this research.

## Supplementary Materials

The following supporting information can be downloaded at: https://www.mdpi.com/article/10.3390/ijms27135958/s1.

## Abbreviations

CD8^+^ T, cluster of differentiation 8 positive T cell; Tex, exhausted CD8^+^ T cell; Teff, effector CD8^+^ T cell; Tcm, central memory T cell; Tem, effector memory T cell; Tregs, regulatory T cells; TME, tumor microenvironment; ICB, immune checkpoint blockade; PD-1, programmed cell death protein 1; *CTLA4*, cytotoxic T-lymphocyte-associated protein 4; LAG-3, lymphocyte activation gene 3; TIM-3, T-cell immunoglobulin and mucin-domain containing-3; 2B4, CD244 molecule; *GPR171*, G-protein-coupled receptor 171; GPCR, G-protein-coupled receptor; *CXCR4*, C-X-C motif chemokine receptor 4; NK, natural killer cell; scRNA-seq, single-cell RNA sequencing; GEO, Gene Expression Omnibus; NCBI, National Center for Biotechnology Information; PBMC, peripheral blood mononuclear cell; CRC, colorectal cancer; ESCA, esophageal carcinoma; KIRC, kidney renal clear cell carcinoma; NPC, nasopharyngeal carcinoma; NSCLC, non-small cell lung cancer; Seurat, single-cell analysis R package; Harmony, batch correction algorithm for single-cell data integration; SingleR, automated cell type annotation method; COSG, cell marker gene identification tool; DEG, differentially expressed gene; FDR, false discovery rate; GO, Gene Ontology; BP, biological process; hdWGCNA, high-dimensional weighted gene co-expression network analysis; TOM, topological overlap matrix; Monocle, trajectory inference algorithm for single-cell data; MNN, mutual nearest neighbor; pySCENIC, single-cell regulatory network inference and clustering; GRN, gene regulatory network; AUCell, gene set activity scoring method; scTenifoldKnk, single-cell virtual gene knockout framework; PCA, principal component analysis; UMAP, uniform manifold approximation and projection; HVG, highly variable gene; PKA, protein kinase A; CREB, cAMP response element-binding protein; *CREM*, cAMP response element modulator; AP-1, activator protein 1; *JUN*, Jun proto-oncogene; *NR4A2*, nuclear receptor subfamily 4 group A member 2; *FABP5*, fatty acid-binding protein 5; *DUSP4*, dual specificity phosphatase 4.
