# Supplementary material for: Single-Cell Pan-Cancer Atlas Reveals GPR171 as a Candidate Marker of CD8+ T-Cell Dysfunction
Source: Int J Mol Sci. 2026 Jul 2;27(13):5958. doi: 10.3390/ijms27135958 (PMC13361737; doi:10.3390/ijms27135958)
Supplement: Supplementary file 1 [file ijms-27-05958-s001.zip › ijms-4370621-supplementary/supplementary figures.pdf]

Supplementary Materials

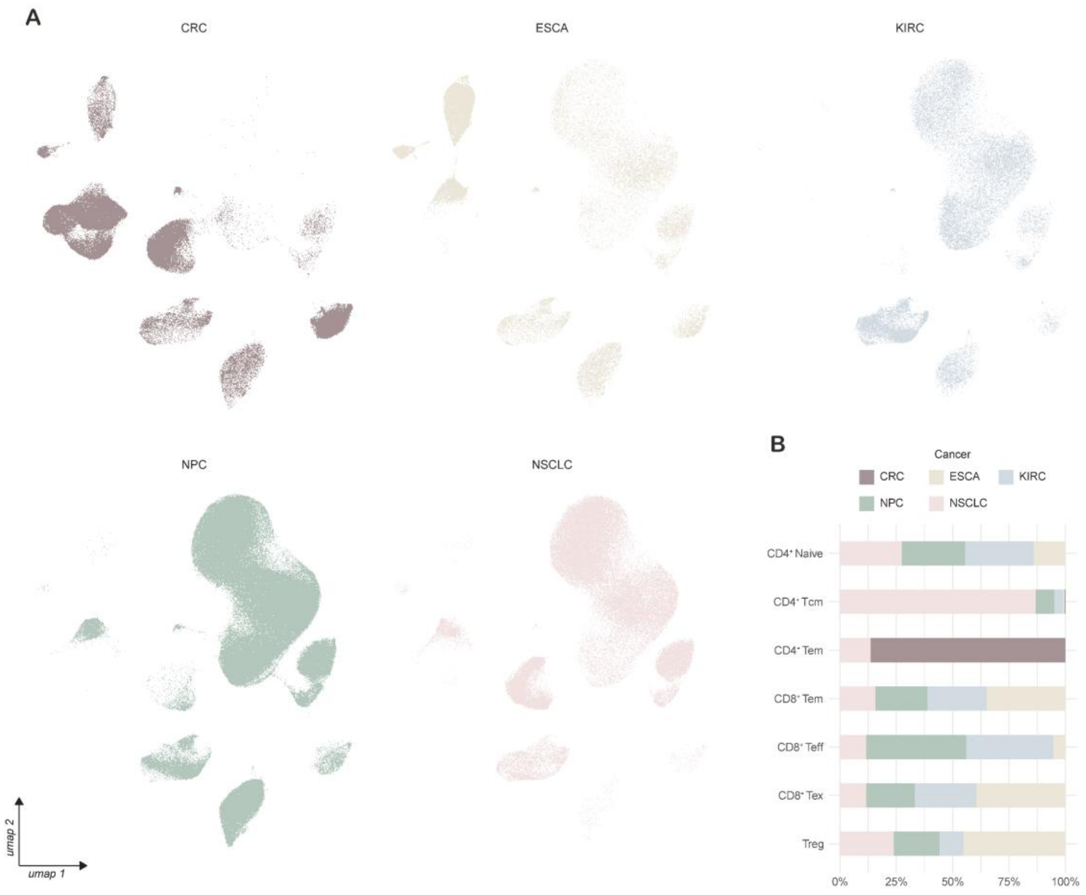

Figure S1. Heterogeneity of T cell subsets across different cancer types. A: UMAP visualization of cells from different cancer types, colored by cancer type. B. Bar plot showing the proportional distribution of cancer types within each T cell subset.

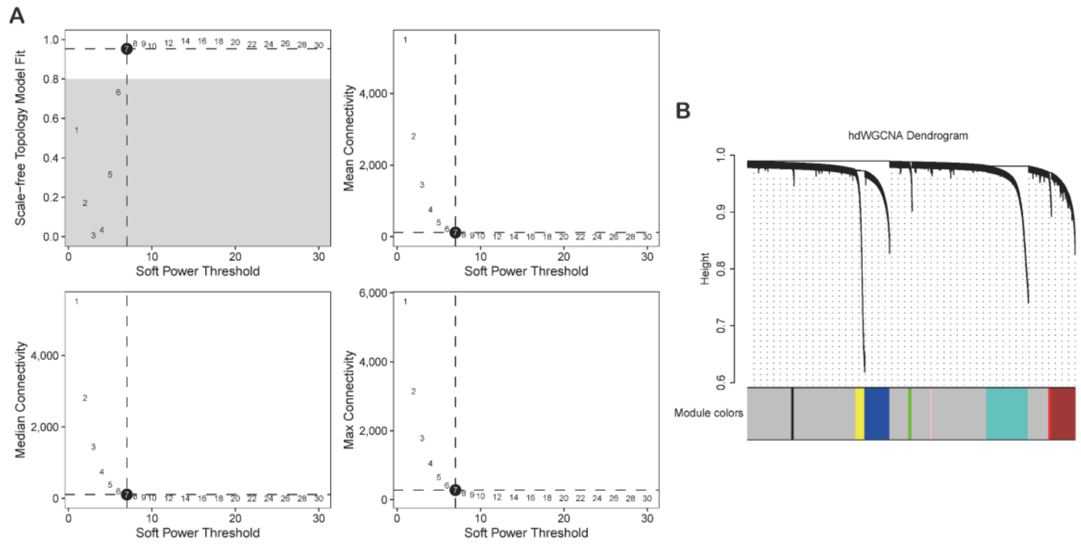

Figure S2. hdWGCNA analysis of CD8⁺ Tex and CD8⁺ Teff cells. A. Selection of the soft-thresholding power for hdWGCNA. Plots show scale-free topology model fit, mean, median, and maximum connectivity across a range of soft power thresholds; the chosen threshold (7) is indicated. B. Dendrogram of gene modules identified by hdWGCNA. Modules are color-coded.

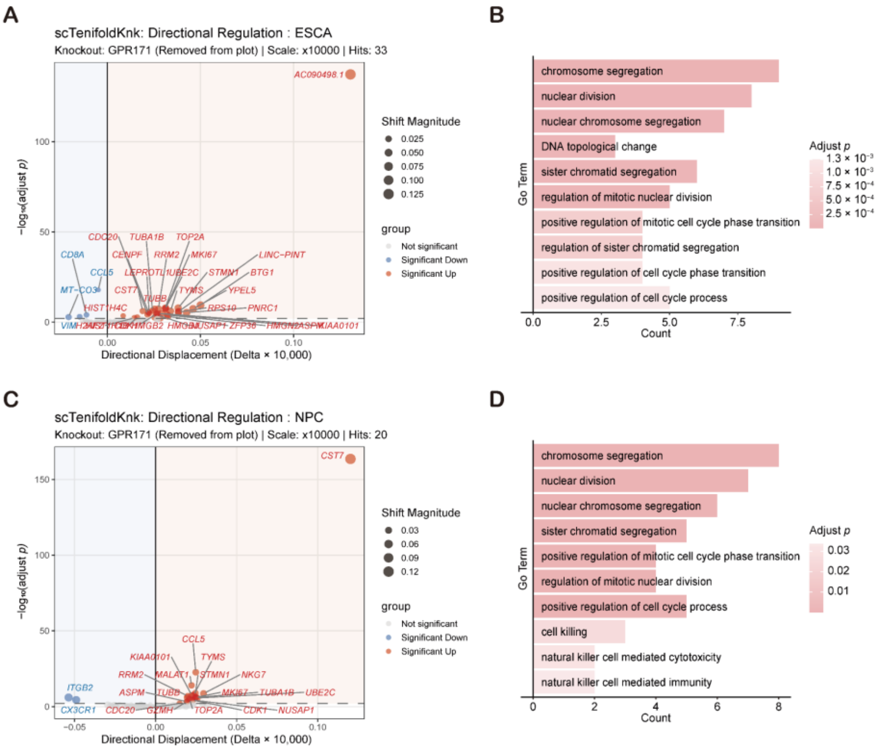

Figure S3. scTenifoldKnk analysis of CD8⁺ Tex cells in ESCA and NPC cohorts. A. Volcano plot showing differential gene expression changes induced by virtual knockout of GPR171 using scTenifoldKnk in ESCA samples, with genes shifted upon perturbation highlighted. B. GO-BP enrichment analysis of genes upregulated after GPR171 virtual knockout in ESCA samples. C. Volcano plot showing differential gene expression changes induced by virtual knockout of GPR171 using scTenifoldKnk in NPC samples, with genes shifted upon perturbation highlighted. D. GO-BP enrichment analysis of genes upregulated after GPR171 virtual knockout in NPC samples.
